# The Missing Link Between Memory and Reinforcement Learning

**DOI:** 10.3389/fpsyg.2020.560080

**Published:** 2020-12-10

**Authors:** Christian Balkenius, Trond A. Tjøstheim, Birger Johansson, Annika Wallin, Peter Gärdenfors

**Affiliations:** ^1^Lund University Cognitive Science, Lund University, Lund, Sweden; ^2^Palaeo-Research Institute, University of Johannesburg, Johannesburg, South Africa

**Keywords:** memory model, decision making, accumulator model, episodic memory, semantic memory

## Abstract

Reinforcement learning systems usually assume that a value function is defined over all states (or state-action pairs) that can immediately give the value of a particular state or action. These values are used by a selection mechanism to decide which action to take. In contrast, when humans and animals make decisions, they collect evidence for different alternatives over time and take action only when sufficient evidence has been accumulated. We have previously developed a model of memory processing that includes semantic, episodic and working memory in a comprehensive architecture. Here, we describe how this memory mechanism can support decision making when the alternatives cannot be evaluated based on immediate sensory information alone. Instead we first imagine, and then evaluate a possible future that will result from choosing one of the alternatives. Here we present an extended model that can be used as a model for decision making that depends on accumulating evidence over time, whether that information comes from the sequential attention to different sensory properties or from internal simulation of the consequences of making a particular choice. We show how the new model explains both simple immediate choices, choices that depend on multiple sensory factors and complicated selections between alternatives that require forward looking simulations based on episodic and semantic memory structures. In this framework, vicarious trial and error is explained as an internal simulation that accumulates evidence for a particular choice. We argue that a system like this forms the “missing link” between more traditional ideas of semantic and episodic memory, and the associative nature of reinforcement learning.

## 1. Introduction

Vignette 1: Pat is visiting Sam for the first time in her country home. Pat loves searching for mushrooms, in particular chanterelles. Sam knows nothing about mushrooms, but she has heard that there are chanterelles in a nearby forest so she offers to take Pat there. When they arrive, Pat starts looking around for suitable biotopes. To the left, there are some alder trees so Pat immediately knows that the ground there is too wet for chanterelles. To the right, there is a spruce plantation and that is normally too dark for chanterelles. But straight ahead is an open beech forest with dry leaves on the ground. So Pat heads in that direction.

Vignette 2: Sam is arriving in her car late in the evening to a small town in France and is looking for a hotel. Her mobile phone is out of battery and the car does not have a GPS. The streets are narrow and winding and there is nobody to ask. Suddenly she remembers the old rule of thumb “Cherchez léglise”—search for the church—and as she sees the church tower now and then from the streets, she manages to find the church. Close to the church there is a hotel.

This article is about how memories from earlier events may influence choice tasks. The central idea is that memories induce correlations in the form of semantic and episodic associations that may be useful in a new choice situation. From earlier mushroom expeditions, Pat has learned about correlations between the type of vegetation and the likelihood of finding different kinds of mushrooms. Such correlation is part of the semantic memory. Sam knows from experiences of other small towns that it is probable that there is a hotel close to the church, a form of episodic memory. She may even explicitly remember a previous episode from a specific hotel and its proximity to a church. In decision making, humans and other animals frequently exploit knowledge of correlations that they have learnt from earlier, similar problems.

Reinforcement learning is one of the major models of how to act in an environment so that reward is maximized. There are two main components in a standard reinforcement learning system (Sutton and Barto, 2018). The first is a component that estimates the value of an action in a particular state. This component can be a simple table, as in the original Q-learning algorithm for reinforcement learning (Watkins and Dayan, 1992), or some form of function estimator that is able to compute the value also in new states and for new actions based on their similarity to previously trained states and actions (Baird, 1995; Xu et al., 2014). The second component is a decision mechanism that selects a particular action depending on the estimated value of the different actions. The simplest strategy is to always select the action with the largest value, but in order to promote exploration and learning, it is necessary to at least sometimes select other actions where the value is not known or is uncertain. Regardless of what decision mechanism is used, the reinforcement learning framework assumes that the value of an action is a function of the present state and the possible actions. This may be sufficient to explain routine decisions but in general, we often collect evidence for different alternatives over time and take action only when sufficient evidence has been accumulated (Ratcliff et al., 2016). Such a strategy can be seen both in humans and in animals.

Consider a situation where we have to choose between two products, two packages of pasta, in the store. Each package has a number of visual features such as shape, size, color, price tag, and branding that together suggest the properties of its content. There is even a list of ingredients in small print that may give additional information. The value of the item is not available directly but results from a process that integrates the different pieces of information on the package. As we scan the different alternatives, we gradually get a picture of which item to choose. In simple cases, each visible attribute of the package may add to the evaluation in a direct way. However, in most cases, we need to consult our semantic memory to make a more informed decision. We may associate the pasta brand name with other groceries that we have bought previously, like tomato sauce, or Parmesan cheese; or we may come to think of other properties related to the packaging of the pasta. For example, colorful cardboard boxes may associate to a fancy Italian restaurant, and so to better quality pasta than a simple plastic packaging. Through our semantic memory, experiences with other related groceries will influence our evaluation of the packages on the shelf. Even the location of the item on the shelf, how hard it is to reach, or whether the shelf is full or not, may influence the decision.

Decisions can also take into account our previous experiences stored as episodic memories. We may recall our earlier experience with this brand. Perhaps we recall that the last time we bought a product in a similar package, it was very hard to open, or maybe we remember eating this particular item as part of a fantastic dinner. Such recalled episodes contribute to the evaluation in a positive or negative way. Episodic memories can also be used to forecast what will happen in the future (Atance and O'Neill, 2001; Hassabis et al., 2007; Schacter et al., 2017). We may imagine combining the item in front of us with something in the fridge at home.

We want to propose that decisions like these are made not by direct evaluation of the item in front of us, but by imagining a future where we have made a particular choice (Atance and O'Neill, 2001; Schacter et al., 2017). It is this future state that is evaluated, rather than the direct properties of the item. This contrasts with standard reinforcement learning that is not forward looking in this way. Instead, decisions made by a reinforcement learning model depend on the estimated value having propagated backwards from the final experienced rewarding state and this requires repeated testing of many successive decisions leading to the eventual goal.

In the model we propose, the future state may never have been experienced and can potentially be imagined for the first time during the decision-making process (See Balkenius et al., 2018). Our proposal may superficially look like a planning process, but is fundamentally different. It is not used to test a sequence of actions and evaluate the result, rather, the decision process imagines different future states and uses the evaluation of those to make a decision about a choice here and now. It is not necessary to come up with all the steps needed to reach the imagined future state, but the choice we finally make should be critical to that future state for the decision to be useful. That is, it should make a discernible positive difference. This means that we may also decide that it does not matter which particular choice is made. Hence in the example with shopping for an Italian dinner, we may conclude that the particular brand of pasta we buy is not important. There is no qualitative difference in projected value between scenarios involving different pasta types. The sauce may make a much larger difference, and so we can choose the cheapest pasta and reserve money for the sauce instead. This would be a case of satisfying rather than optimizing in decision making (Simon, 1972).

One can find extensive evidence from both psychology and neuroscience for the type of mechanisms we propose. Firstly, there is a long history of sequential decision making models in psychology (Usher and McClelland, 2004; Mather and Sutherland, 2011; Johnson and Ratcliff, 2014; Mather et al., 2016; Ratcliff et al., 2016; Evans and Wagenmakers, 2019), but they have mostly been applied to the type of immediate choices outlined above and not to choices based on memory processes. Their main component is an accumulator that collects evidence for different alternatives until a decision criterion is reached.

Secondly, in computational neuroscience, the idea of neural competition is a cornerstone of many theories of brain function (Amari, 1977; Grossberg et al., 1978; Erlhagen and Schöner, 2002). For decision making, such competition mechanisms are central to making choices and below we outline an accumulator model that includes several types of competition mechanisms. Of particular interest are leaky competing accumulator models that incorporate aspects of both the psychological and neurophysiological models (Usher and McClelland, 2001, 2004; Johnson and Ratcliff, 2014). Such competitive processes in the brain are modulated by arousal that can make the competition more or less random (Aston-Jones et al., 1999; Usher et al., 1999; Mather et al., 2016) and shift between exploration and exploitation (Gilzenrat et al., 2010).

In addition to including memory processes in a decision model, we also want to address how attention is used both for perceptual selection and as an indexing system into the accumulator component of the model. The currently attended spatial location of the attention system is used to index separate accumulators that are associated with different spatial locations. Empirical evidence suggest that humans use spatial locations as hooks for working memories to store information about a particular stimulus or object (Richardson and Spivey, 2000). This is a form of deictic coding that allows different choices to be equated with different spatial locations (Ballard et al., 1997).

Our aim in this paper is to show how a memory mechanism that handles episodic and semantic memories can be combined with a decision mechanism to make choices based on perceptual features. We present a computational model where we combine a perceptual system that can scan different choices while using a memory component to process both semantic and episodic associations that help evaluate the different choices. A spatial attention component directs attention to the different choices and is also used to index different value accumulators that add up evidence for each alternative. The system-level computational model demonstrates how perception, attention, memory, and choice mechanisms can interact in decision-making processes. The focus here is on the interaction of these components rather than on learning of values or on initial storage in memory. In particular, the simulated model described below contains only pre-set associations.

## 2. A System-Level Model of Memory Based Decision Making

System level models of the brain aim at explaining which different components are needed for a particular cognitive function. They aim at answering a number of questions about the architecture behind an ability (Balkenius et al., 2010): Which are the required components and what are their functions? How do they interact? What information is transferred between the components and which is it coded? An overreaching assumption of system level modeling is that it presents the overall organization of the different components and their dynamic interactions that determine many of the properties of the system. Many of these properties will be present even when each of the components are modeled in a minimal way. The model we present here is based on this assumption in the sense that each component is as simple as possible to exhibit the desired properties.

**Figure 1:**
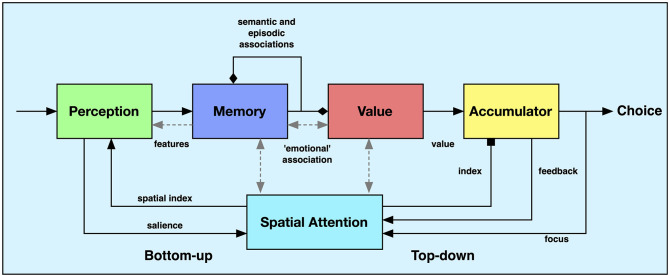
Overview of the proposed model. The system-level model combines components for perception, memory, valuation, value accumulation, and spatial attention. The black arrows represent non-learnable connections between the components. The diamond arrowheads represent feedback and forward associations that have been previously learned. The square arrowhead represents a facilitating input, in this case the selection of a particular accumulator. The gray arrows represent interactions that we do not address in this paper. See text for further explanation.

The model consists of five main components (Figure 1). The first is the perceptual system that produces a sequence of “feature descriptors” from an attended object. Second, a memory system receives these feature vectors and generates associations from them, including direct “emotional” associations coding for value, semantic associations to similar or associated stimuli, and episodic associations that are used to imagine future states. Third, a value system is activated when “emotional” associations are triggered and produces value estimations for each memory state. These are finally accumulated in the fourth component until a decision criterion is met and the system produces a choice as output. There is also a spatial attention system that is responsible for directing attention to the different choices, indexing the accumulators based on the current spatial attention, and for locking on to the chosen object. A more formal description of each of the components is presented below.

### 2.1. Perceptual Scanning

The perceptual system transforms a visual input into a sequence of feature vectors that describe the perceived scene. As the perceptual system attends to a particular object, different feature vectors are produced over time as different attributes of the objects are processed. Note that we use the word “feature” for the individual elements of the perceptual feature vectors rather than as synonyms for attributes or properties.

**Figure 2:**
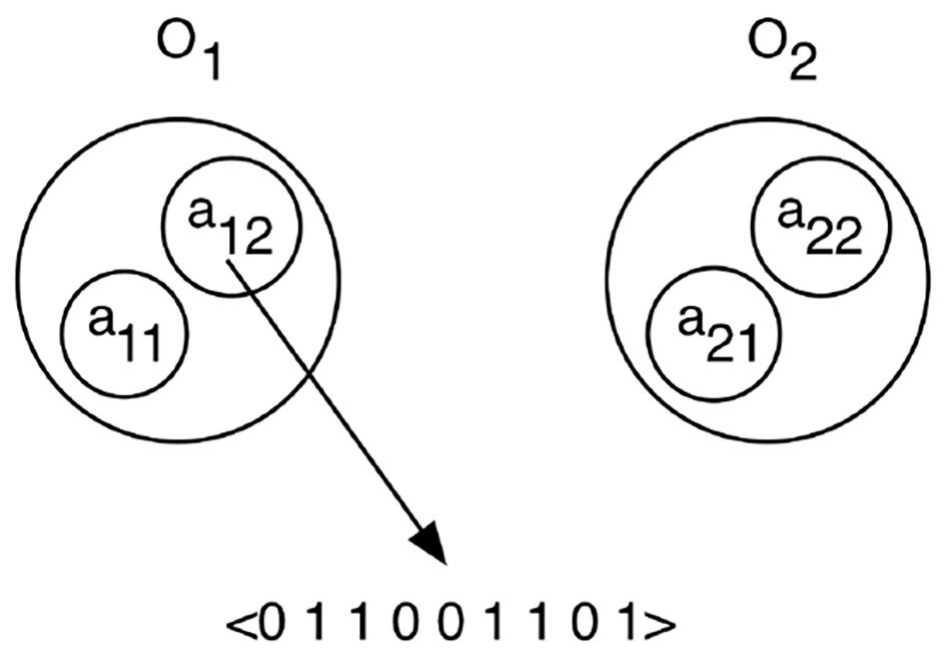
The observed scene is modeled as a set of objects *O*_*i*_ that each has a number of attributes *a*_*ij*_. When an attribute is attended, it will produce a binary feature vector that describes the attribute.

Each object is considered to contain several attributes that can be perceived and each attribute gives rise to a particular feature vector when perceived. An object *O*_*i*_ is modeled as a set of attributes {*a*_*ij*_} where each attribute is associated with a binary feature vector of size n, *a*_*ij*_ = 〈 *f*_*ij*1_ … *f*_*ijn*_〉. Figure 2 shows a schematic image of how two objects are coded.

In the minimal case, attention is randomly directed to the different objects, but the model also allows for attention to be based on perceptual salience through the bidirectional connections with the spatial attention component. Furthermore, top-down attention from the accumulators can also bias attention toward the hitherto most valued object and finally lock attention to the selected object.

In the model that is presented below, attention is directed to one object at a time, and during that time, the perceptual system is allowed to randomly process each attribute of the object for a number of simulation cycles, allowing the memory system to produce a sequence of associations from the processed attribute.

### 2.2. Memory

**Figure 3:**
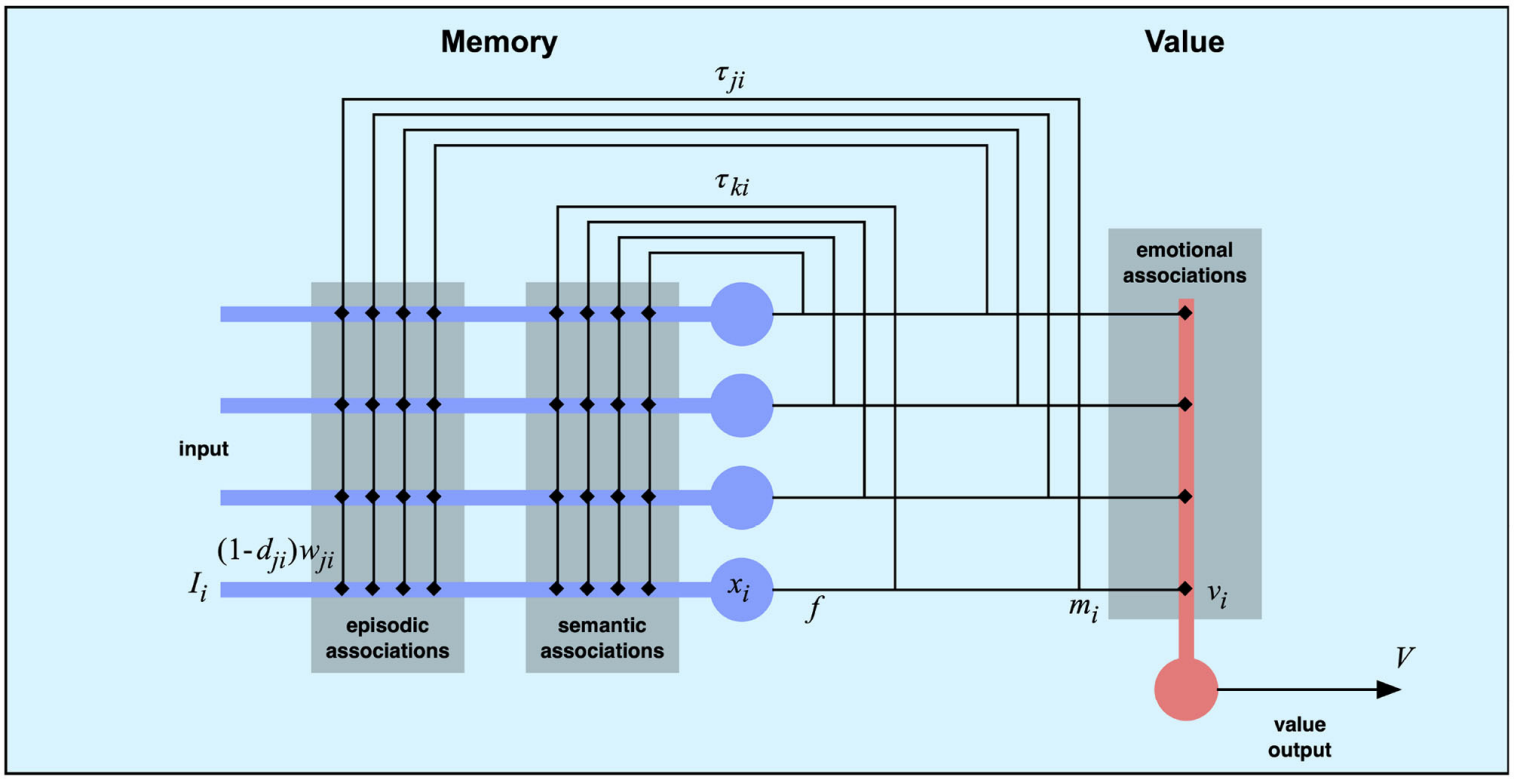
The memory and value components. A recurrent connectionist network uses two types of connections to implement semantic and episodic memory. The semantic associations are fast and allow the network to settle in attractor states. The episodic associations have a longer time constant τ that makes the network jump between states.

The second component of the model is the memory system (Figure 3). This component takes the current feature vector from the perceptual system as input and produces sequences of memory states based on previously learned associations. We base the memory component on an earlier memory model (Balkenius et al., 2018). However, here we use a unified memory state rather than distinguishing between the “what” and “where” systems of the earlier model. The memory associations can be of one of three types.

The first can be called “emotional” or “value” associations. These produce the output from the memory that is sent to the value component and then on to the accumulator stage. For the purpose of making choices, these are the associations that determine which choice will eventually be made.

The second association type is used for low latency associations that allow the memory system to reach attractor states as usual in recurrent networks (Hopfield, 1982). To produce semantic memory transitions we assume that synaptic depression limits the time the memory state stays at an attractor (Abbott et al., 1997; Tsodyks et al., 2006). Once the effect of synaptic depression kicks in, the attractor will collapse and transition to a semantically related state and another attractor. This is sometimes called latching dynamics (Lerner et al., 2010; Aguilar et al., 2017) and is the mechanism of free association.

Associations of the third type have a longer latency and they produce episodic memory transitions (Herrmann et al., 1993). The basis for this mechanism is a delay imposed on the recurrent connections of the episodic memory (Sompolinsky and Kanter, 1986). This forces the memory state out of the current attractor and into a predicted future state.

Considering the case with a single delay τ_*ji*_ for each recurrent connection, the state *x* of the memory network is governed by the following equation, where *I* is the input, *w* are the weights of the connections, *d*_*ji*_ is the synaptic depression and *f* is the activation function of the nodes.

(1) $$\begin{array}{r} \frac{d}{dt}x_{i}\left(t\right)=I_{i}\left(t\right)+\left(1-d_{ji}\right)w_{ji}\sum_{j}f\left(x_{j}\left(t-\tau_{ji}\right)\right) \end{array}$$

Synaptic depression is assumed to increase as a function of the signal flowing through the corresponding connection (Lerner et al., 2010; Aguilar et al., 2017; Balkenius et al., 2018). The processes of the memory system are also influenced by modulating signals from arousal systems that can determine the level of randomness of the state transitions (Aston-Jones et al., 1999; Chance et al., 2002; Aston-Jones and Cohen, 2005). For simplicity, we have not included these inputs in the equations here. A more detailed description of this memory component can be found elsewhere (Balkenius et al., 2018).

Here, we do not consider learning in the memory system but assume that this has happened earlier. We are only concerned with the retrieval of previously stored associations and how they influence the decision process. For the purpose of this paper, the important aspect of the memory model is that any input will produce a sequence of memory states, each of which may or may not be associated with value. It is this indirect connection between perceptual input and value accumulation that is responsible for the properties of the model described in section 4. Another useful property of the memory model is that it can not only recall earlier episodes, but also produce new combinations of previous memories using random transitions between similar memory states (Balkenius et al., 2018). We do, however, not explore this feature of the memory system further.

### 2.3. Value

The value component is responsible for associating memory states with value and is the first stage of the actual evaluation process. As the memory system transitions through a sequence of states, the value system calculates the value of each state and sends the result to the accumulator described below. Each memory state vector *m*, where *m*_*i*_ = *f*(*x*_*i*_), is associated with a scalar value *V* through a linear mapping *v*, that is,

(2) $$\begin{array}{r} V=vm^{⊺}. \end{array}$$

The vector *v* contains the value for each of the elements of the memory state. This function is similar to the value function in reinforcement learning when a linear function approximation from a binary state representation is used (Xu et al., 2014). The values can be learned through classical conditioning (Rescorla and Wagner, 1972; Balkenius and Morén, 1998), often expressed in the form of TD-learning (O'Doherty et al., 2003; Sutton and Barto, 2018).

However, since value is used here in a sequential accumulation process (described below), it is not necessary that the value component supports higher order conditioning, which is otherwise the basis for chaining in reinforcement learning. A simple learning rule, like the delta rule (Widrow and Hoff, 1960) is sufficient for the model to train on external reinforcers. Note that we do not claim that higher order conditioning does not occur, or that value learning by classical conditioning is not more complicated. The present model is indeed compatible with more elaborate models of classical conditioning.

### 2.4. Accumulator

**Figure 4:**
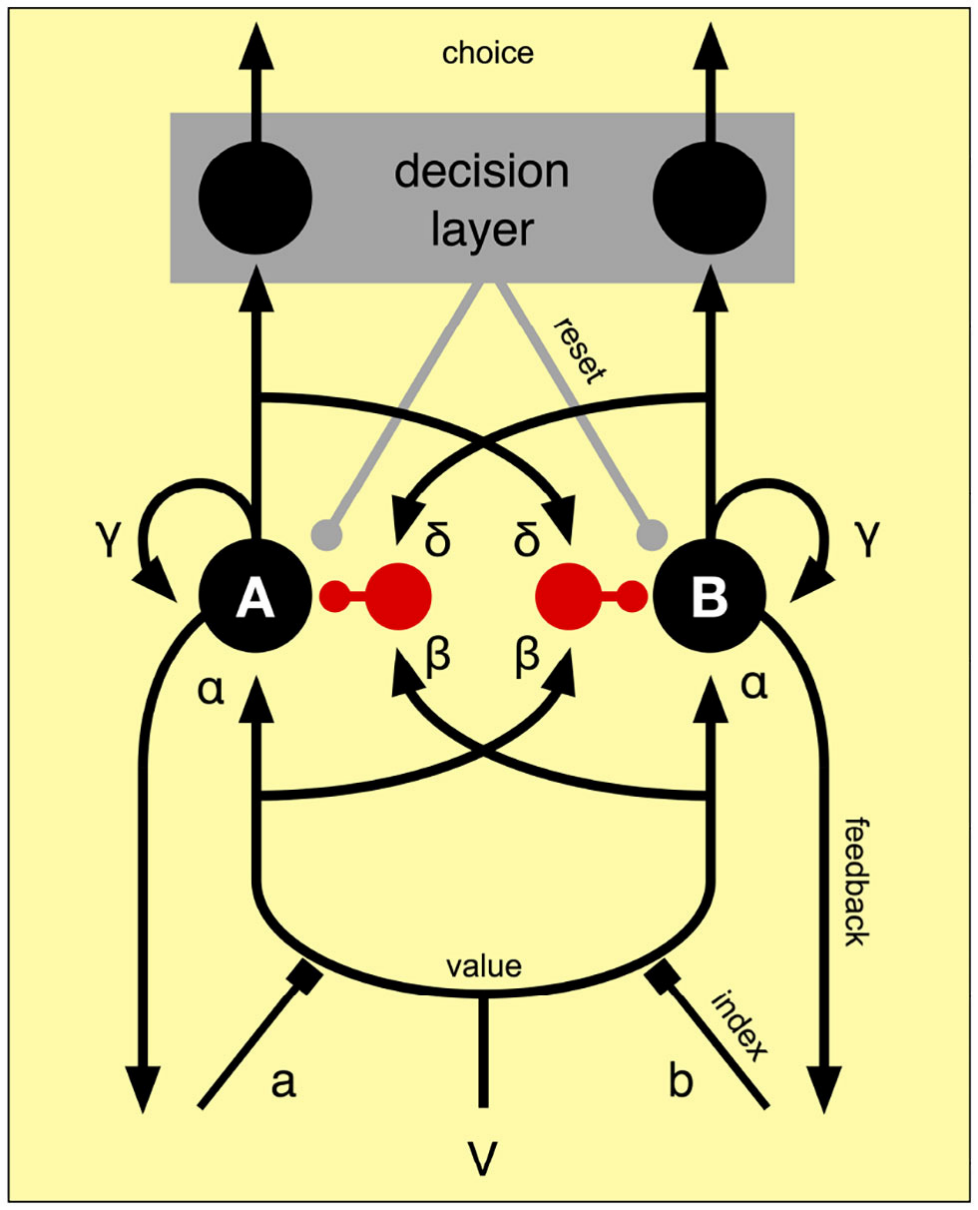
Competition between two accumulators A and B. One of the accumulators is selected by the spatial index a or b by their facilitation of the connections from the value input V (as explained in section 3.3) to the two accumulators. The excitatory value input is weighed by α before it reaches the accumulator. The selected input is also weighed by β before it connects to an inhibitory inter-node (in red). This implements forward inhibition of the accumulators. There is also recurrent excitation weighted by γ and recurrent inhibition weighted by δ. The decision layer detects when one of the accumulators has reached the decision threshold and activates the corresponding output.

The accumulator consists of integrators indexed by spatial attention. These integrate value for the currently attended object. When the integration process reaches a particular criterion, the winning alternative in a decision layer is chosen (Figure 4). By assuming that spatial attention is responsible for selecting the appropriate accumulator, the same model will work for an arbitrary number of objects and it gives spatial attention a central role in decision making.

The model allows for four types of connections weighed by individual gains: forward excitation (α), forward inhibition (β), feedback excitation (γ), and feedback inhibition (δ). The state of the accumulators is called *x* and the dynamics of the model is controlled by,

(3) $$\begin{array}{r} \frac{d}{dt}x_{i}=-\lambda x_{i}+\alpha I_{i}+\underset{j\neq i}{\sum }\beta I_{j}+\gamma f\left(x_{i}\right)+\underset{j\neq i}{\sum }\delta f\left(x_{j}\right)+N\left(\sigma \right) \end{array}$$

where *I*_*i*_ is the value from the value component when *i* is the accumulator selected by the input from the spatial attention system, and 0 otherwise. λ is a decay constant and *N*(σ) is a normally distributed noise term. *f* is the activation function of the nodes (ReLU). The decision layer consists of a winner-takes -all network that only reacts once one of the accumulators reaches its decision threshold. For all simulations reported below, a fixed value of 1 was used. Once the threshold is reached, the decision layer will reset the accumulators.

### 2.5. Spatial Attention

The spatial attention system is responsible for shifting attention to each object. It is controlled by bottom up salience as well as top-down feedback from the decision mechanisms and selects which object is attended. It is also used as a spatial index in the memory system and to select the appropriate accumulator for each choice.

The activity of the accumulators can be made to influence the selection in the attention component. In this case, the complete system will allocate more time to the alternative that looks best so far in the evaluation. As a consequence, it is more likely to win the competition and will also do so more quickly. The probability of switching attention to stimulus *O*_*i*_ at location *i* at each time step is

(4) $$\begin{array}{r} p_{i}=\frac{\epsilon \left(s_{i}+\varphi g_{i}^{n}\right)}{\underset{j}{\sum }\left(s_{j}+\varphi g_{j}^{n}\right)} \end{array}$$

where *s*_*i*_ is the salience input from the perceptual system and *g*_*i*_ is feedback from the decision process. The parameter φ controls the influence on the attention of top-down feedback and *n* sets the contrast enhancement between the different alternatives. In the simulations reported below, the *s*_*i*_ are set to 0 or 1 to indicate the presence of an object at the corresponding location, but could in principle code for the visual salience of each object. The constant *n* is here set to 2. The parameter ϵ sets the base rate for attentional shifts.

## 3. Properties of the Model

In this section we present a number of computer simulations of the model. It was implemented using the Ikaros framework for system-level brain modeling (Balkenius et al., 2010, 2020). The purpose of the simulations is to illustrate the properties of the system model in different situations rather than to find optimal parameters to reproduce any particular empirical study.

### 3.1. Choosing Between Two Objects

Like other models of choice, the model can handle a situation where there are two objects with one attribute each. Going back to our pasta example, this could be choosing between two different pasta shapes from the same manufacturer or brand. Here only the shape of the pasta differs, otherwise the packaging is approximately the same, and one shape is preferred over the other. Another alternative would be selecting a product solely based on price. A lower price may be preferred, or alternatively, a higher price may be preferred based on perceived higher quality, although in reality such a correlation is weak (Faulds and Lonial, 2001). An advantage of the model is that it can handle either case since the value of an object is coded by an association with the value system that can be set up to reflect price as something increasing or decreasing the value of a product. The model does not sample the value of the product directly. Instead it samples one or several attributes of the product that are indirectly associated with a value.

**Figure 5:**
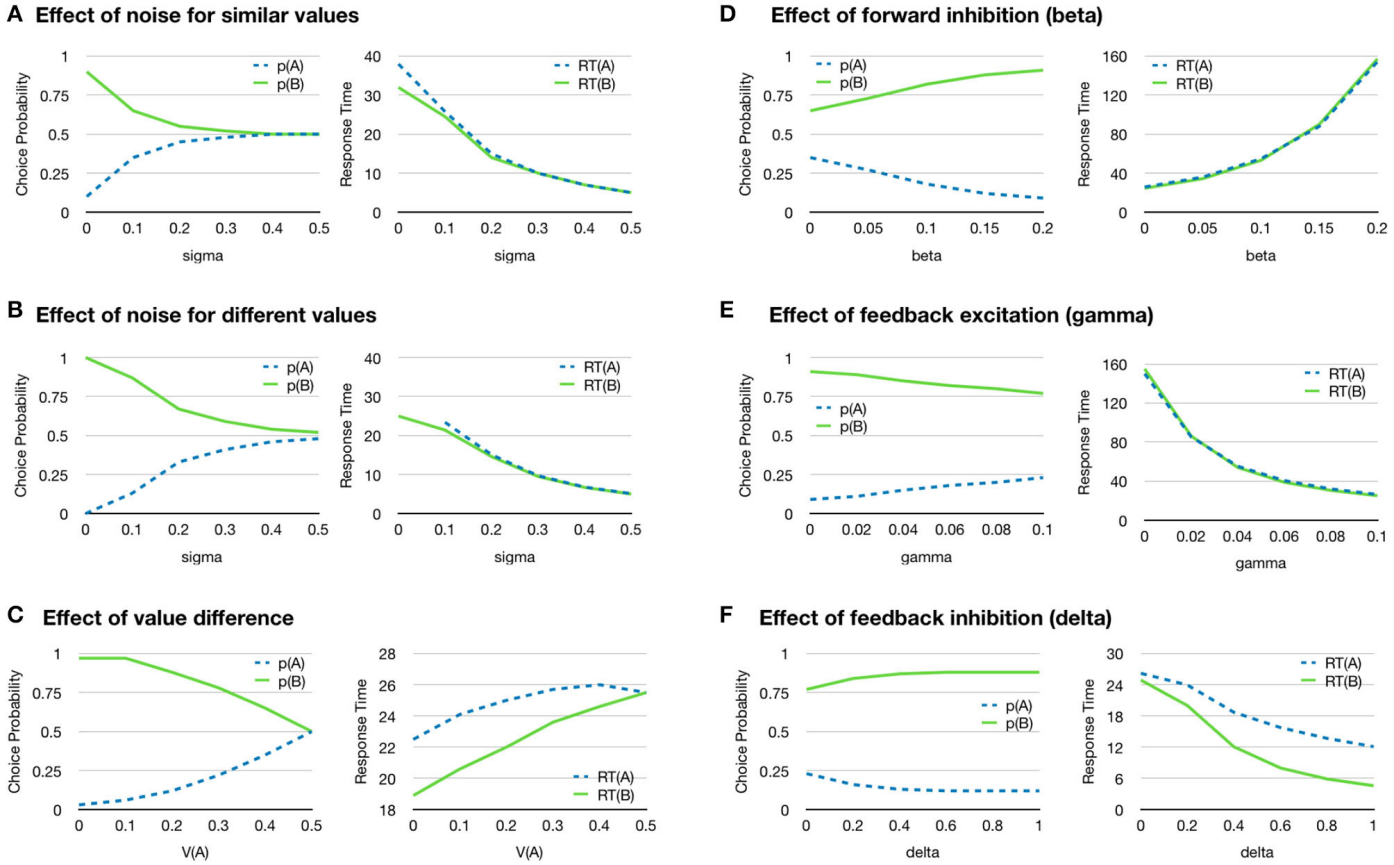
Choosing between two objects with one attribute each. The graphs show the probability of choosing object A or B, and the average response time for each object for different conditions. Since there is only one attribute, the value of the object is given by that single attribute. **(A)** Increased noise (sigma) gives more random choices (left) and faster reaction time (right) for the two objects A and B where the value of A is 0.4 and the value of B is 0.6. **(B)** Increased noise (sigma) gives more random choices (left) and faster reaction time (right) for two objects A and B where the value of A is 0.2 and the value of B is 0.8. **(C)** The probability of selecting each object depends on how different the values V(A) and V(B) are for the two objects. Here values are assumed to sum up to one. The value of A on the x-axis thus represents the similarity of the two objects where V(A) = 0.5 means that the value of both objects are identical. The reaction time increases when the values of the two objects V(A) and V(B) are more similar as the activation of the accumulators takes longer when the values are lower (right). **(D)** Increased forward inhibition (beta) gives slower reaction time and more choices of the alternative with higher value. **(E)** Feedback excitation increases response time and has a smaller effect on choice probabilities. **(F)** Feedback inhibition slightly increases the difference in response probability and reduced response time. All parameters for the simulations are given in the Supplementary Material.

Figure 5 shows some basic properties of the model. By varying the noise level, decisions can be modulated from always taking the one with highest value to fully random selection (Figures 5A,B). An increased noise level also reduces the reaction time.

The difference in value between the two choices influences the process in such a way that a choice between two alternatives that are more similar will take longer time than when their values are very different (Oud et al., 2016) (Figure 5C). It has been observed that many organisms use an excessive amount of time to make decisions between similar alternatives, where too much time is allocated to a choice relative to what is gained by making the correct choice (Oud et al., 2016). In the simulation, the input to each accumulator decreased as the values become more similar, because they are assumed to sum to 1 which makes the decision slower. This can be contrasted with a situation where the preferred alternative stays at 1 while the value of the other is increased. In this case, the reaction time will instead decrease as the total input to the accumulators increases.

The effect of feed-forward inhibition is illustrated in Figure 5D. When this effect is increased, the model is more likely to choose the highest valued alternative, thus, to make a more accurate choice. However, a higher level of feed-forward inhibition will also lead to a longer reaction time. This suggests that the amount of feed-forward inhibition can be used to control a trade-off between accuracy and speed in decision making (Wickelgren, 1977). The explanation for this effect is that increased feed-forward inhibition slows down the accumulation of value and allows the effect of noise to decrease because it is integrated over a longer time. A slower accumulation decreases the probability the decision process will reach the decision threshold as a result of noise.

Feedback excitation has the effect of decreasing response time because it will produce a positive feedback to the accumulators (Figure 5E). Feedback inhibition also decreases the response time, but slightly increases the probability of choosing the more valued object (Figure 5F).

Figures 5D–F can be together considered as showing different phasic aspects of the selection mechanism. Given that accumulator units A and B shown in Figure 4 have some activation threshold, feed-forward inhibition will be more influential before activation occurs, while feedback connections tend to become more dominant after activation. Positive feedback tends to force dynamic systems into quickly settling into new states (DeAngelis et al., 2012) and minimize the transition period, as can be seen in Figure 5E for response time. If considered together with lateral feedback inhibition as in Figure 5F, where competitors are inhibited, the total effect is to enhance contrast between alternatives and optimize response time. Since contrast enhancement is associated with the effect of noradrenaline (NA) (Waterhouse and Woodward, 1980; Usher et al., 1999), this is in agreement with research indicating that NA is involved in decision making. For example, Frank et al. (2001) did simulations on NA deficits in a computational model of ADHD decision making, and found that reaction times became more variable with low phasic NA stimulation. They cite also empirical evidence of higher response time variability in children with ADHD (Leth-Steensen et al., 2000; Castellanos et al., 2005).

It is also possible to change to what extent the model uses later information more than earlier by setting λ lower than one. In this case, the accumulators will leak and ‘forget' earlier input over time (Tsetsos et al., 2010).

**Figure 6:**
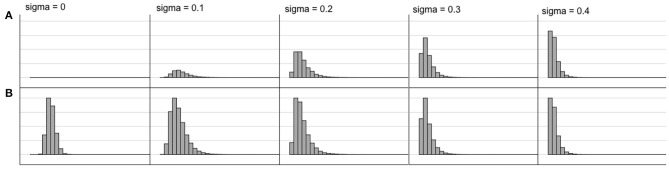
Response time distributions for different noise levels (sigma) for a choice between **A,B** where the value of **A** is 0.4 and the value of **B** is 0.6. Note that the choice distribution as well as the response time distribution changes. For lower noise, the more highly valued alternative is chosen nearly always but with more noise the choices of the two alternatives become more equal and are made more quickly. The noise level thus modulates a dimension from exploitation to exploration.

Figure 6 shows the response time distributions for different levels of noise. These distributions resemble what is empirically found (Ratcliff et al., 2016). In particular, the longer the reaction time, the wider the distribution for the less preferred alternative becomes. Another feature is that choices are made faster but less correctly when the level of noise increases.

### 3.2. Choosing Between Two Objects With Multiple Attributes

Now let us consider choosing between pasta types that are not only differently shaped, but also from different brands. The packaging now differs, as does the price. The main difference compared to the previous case is that the input to the memory component, and subsequently to the value and accumulator components, fluctuates greatly during the evaluation of the two alternatives as attention is moved between the two objects.

**Figure 7:**
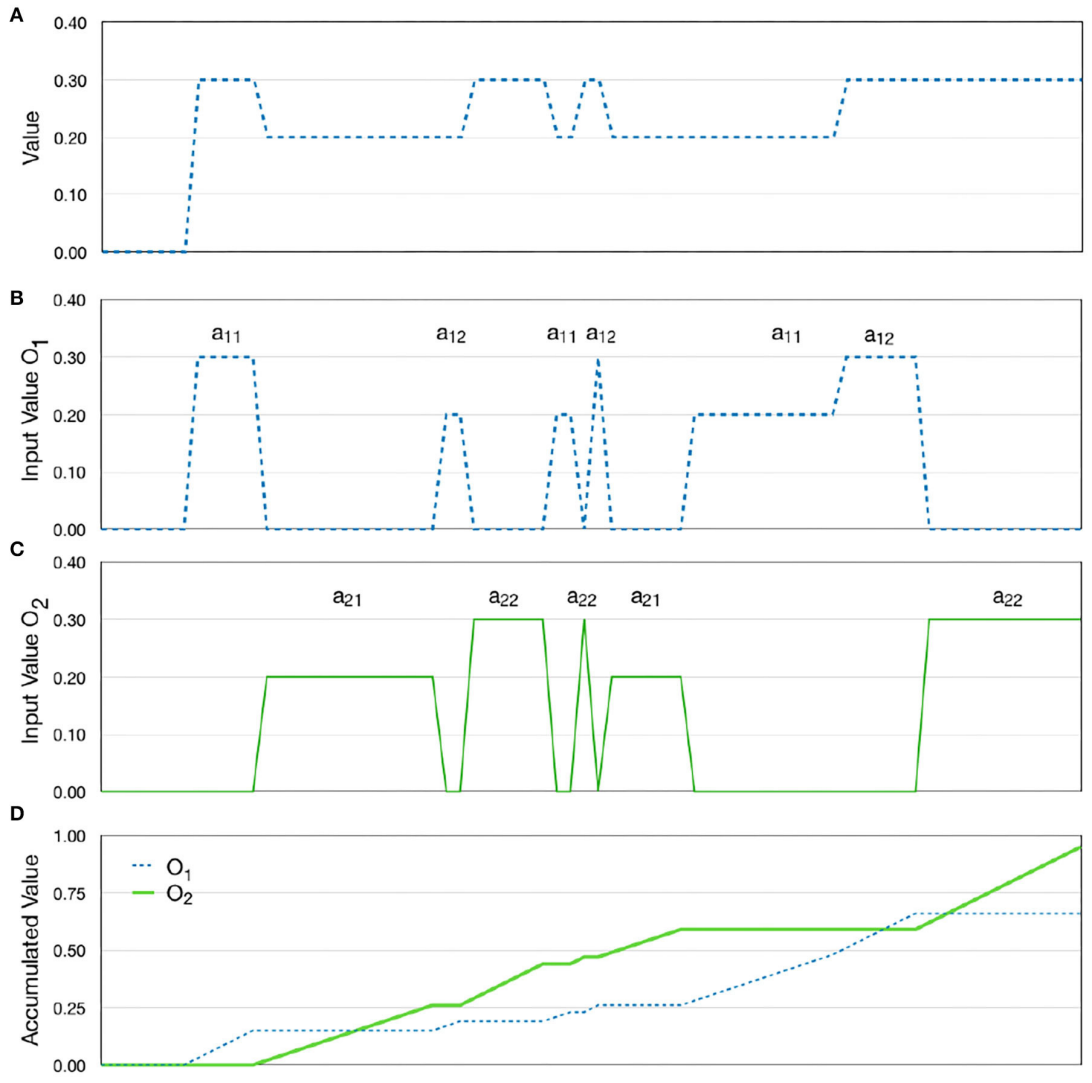
Processing multiple attributes. **(A)** The output from the value component while processing the different attributes of the attended object. **(B,C)** The spatial index from the attention system is used as a selection mechanism that directs the value to one accumulator per object. **(D)** The accumulation of value over time while scanning the different alternatives. (No noise was present to make the plots clearer).

We tested a situation in which alternative A and B both have two attributes. For alternative A, the values for the two attributes are 0.2 and 0.3 while for alternative B, the values are reversed. We tested the model's ability to sum contributions from individual attributes, and as expected the model selected each of the alternatives with probability 0.5 (Figure 7).

### 3.3. Semantic Associations

When the perceivable attributes of an alternative are not associated with any value, we can use our semantic memory to obtain more information about the alternatives. It can also be used to activate associations that in turn may have positive or negative valuations. Let us go back to the example with different pasta shapes from the same manufacturer. One shape is the pipe-like *penne*, while the other is the sea-shell-like *conchiglie*. The latter reminds you of white seashells on an summer beach. This yields pleasurable associations to warmth and relaxation and these positive associations will influence the choice. The two alternatives do not have any immediate evaluation but they associate to situations that do have value. This value is used instead. There may also be other types of semantic associations. Looking at one of the packages you may recall the list of ingredients that you read at an earlier time. The penne is made from durum wheat that you recall as something positive.

**Figure 8:**
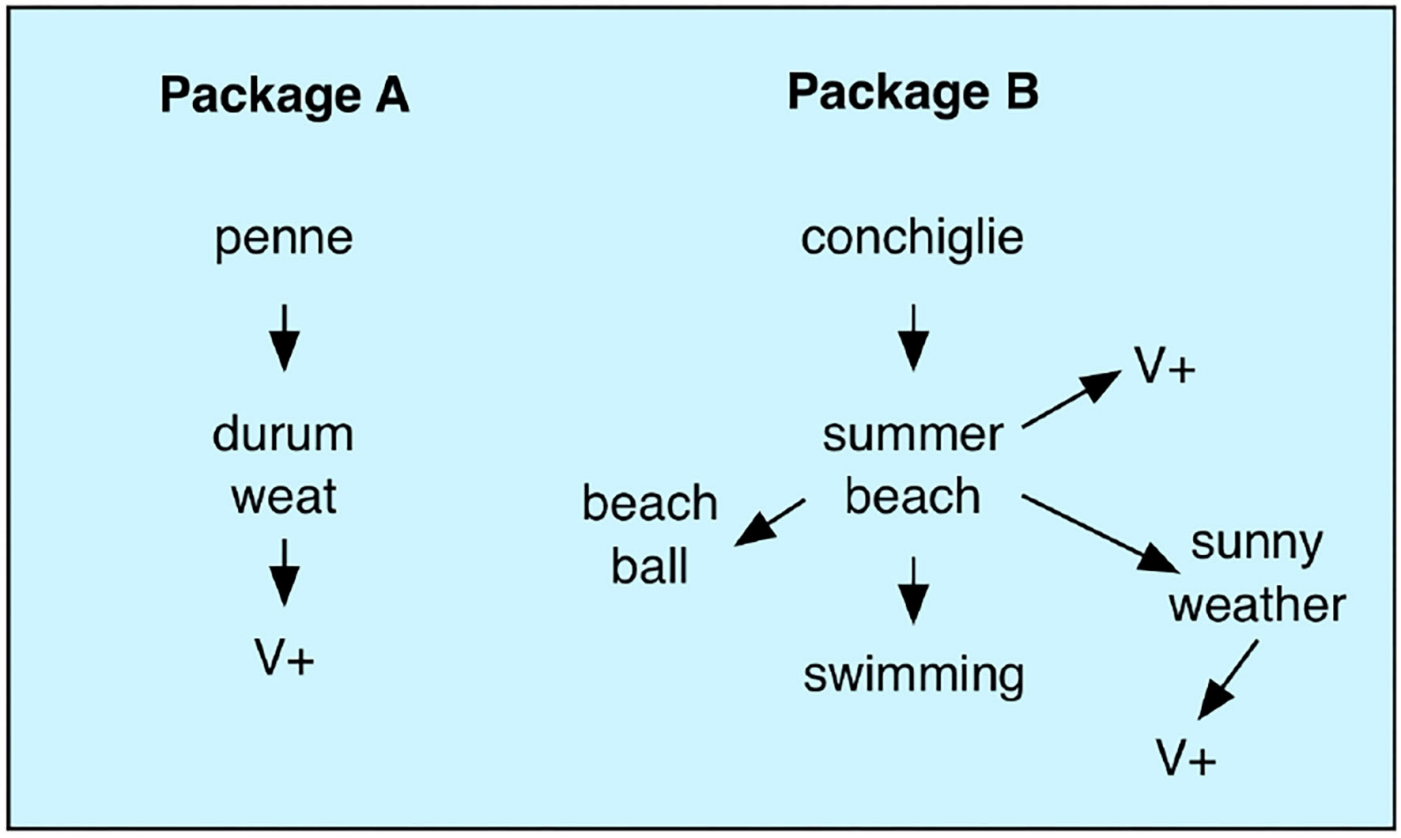
Semantic memory transitions in the valuation process. Looking at a pasta package triggers a chain of semantic associations that may eventually lead to a memory state with value that will influence the decision process. V+ here represents an association with positive value.

Each time you gaze at one of the packages, an associative process will start that make the memory component transition between a number of states (Figure 8). The semantic associations within the memory component makes up a form of semantic network through which the memory state can travel.

In the model, semantic associations depend on two mechanisms. The first is direct low latency associations represented by the *w*_*ji*_ with a low value of τ_*ji*_ in Equation (1). The other mechanism is the transition between semantically similar attractors caused by synaptic depression (*d*_*ji*_ in Equation 1). The details of these semantic memory transitions were described in an earlier paper (Balkenius et al., 2018). Their main feature is that any sensory input will give rise to a sequence of internal memory states that starts from the features of the attended object. Every memory state is potentially associated with a value that will influence the accumulators.

Although free associations can lead memory astray, the memory state will quickly return to relevant states when attention is shifted to the other package, or when a decision threshold is reached. The rather disorganized associative memory can thus support a well-controlled decision process.

### 3.4. Episodic Associations

Continuing with our pasta example, you now happen to see a third pasta shape, the butterfly-like *farfalle*. This particular shape yields fond childhood memories of eating pasta at home where farfalle was the pasta of choice. This is an episodic memory association that may conjure up scenes from your childhood where each part of the scene has its own associations that contribute to the decision. In contrast to semantic memory transitions, the episodic memories play out as learned sequences that reproduce experiences as a sequence of memory states. Each of the states in the sequence can have its own semantic or value associations.

In addition, the episodic associations can also be used to imagine future events after a particular choice was made. You imagine cooking conchiglie while having an amusing discussion about sea shells with you family. The combination of semantic and episodic associations together with noise-induced randomness can produce novel episodic predictions of this kind (Balkenius et al., 2018).

Such a forward looking use of the episodic memory is similar to the forward sweeps found in animal brains as they consider different alternatives (Redish, 2016). The brain activity quickly plays out a possible route through a maze if a particular direction is selected at a choice point. In an influential study, Hassabis et al. (2007) showed that patients with hippocampal lesions impairing episodic memory were unable to usefully simulate future scenarios. In particular, projected scenarios lacked spatial coherence. Together, this indicates the importance of spatial processing and episodic memory in decision making and planning.

**Figure 9:**
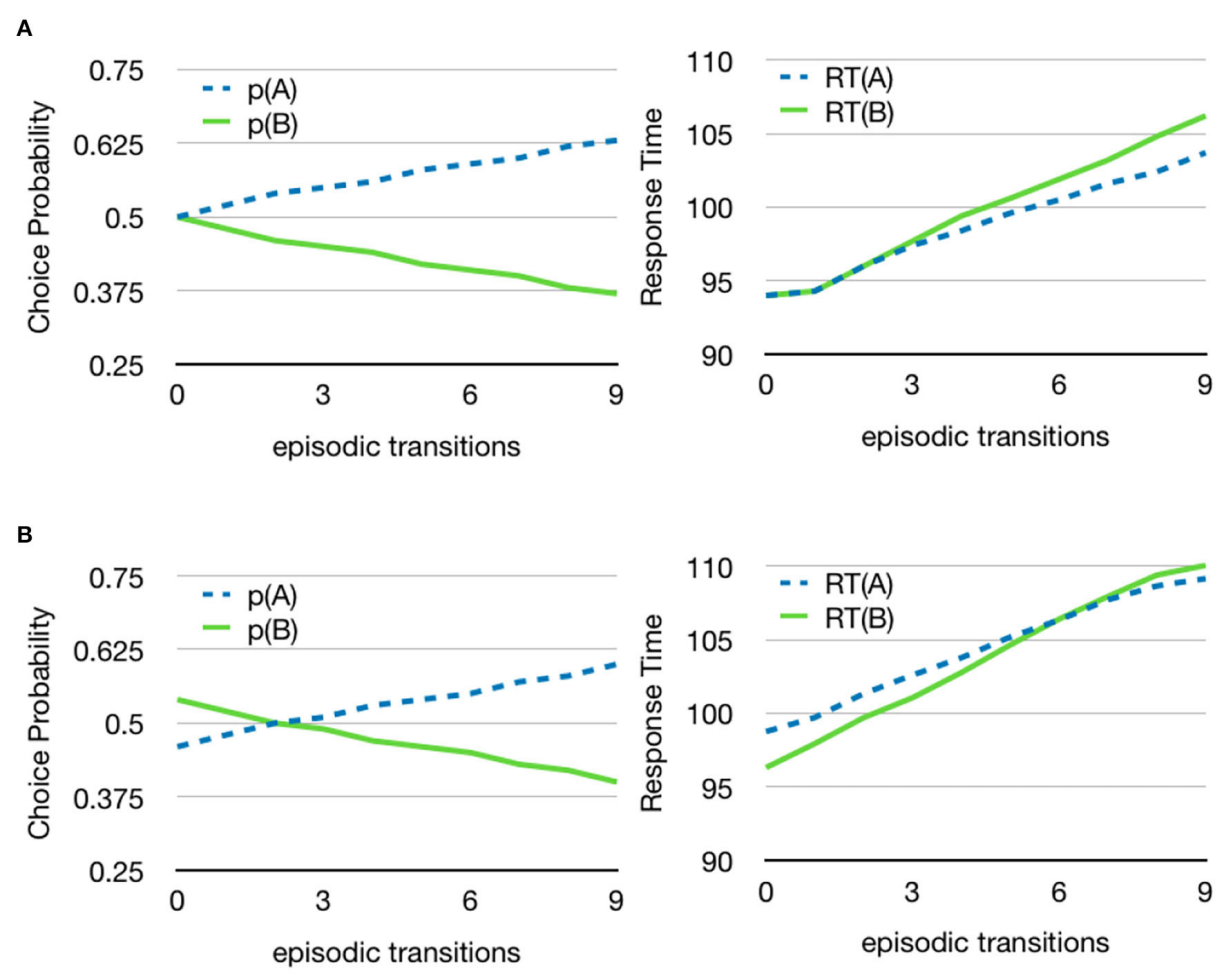
Implicit discounting. Choice probabilities and reaction times for different sequences of episodic transitions. **(A)** A goal that is closer in time is reached through fewer episodic transitions and wins more often. Both goals have value 1. The value for stimulus A is received directly but the value for stimulus B is received only after 0–9 episodic memory transitions. The number of transitions needed to reach a valued memory state depends on how the episodic associations are set up in memory **(B)** Because of the discounting of future value, the model can relate a smaller immediate reward, 0.9 for B, to a future larger reward, 1 for A. For the particular values used in the simulation, the break-off point where the two stimuli are equally valued occurs at two episodic transitions for the higher valued stimulus. See Supplementary Material for additional parameters.

Another aspect of the episodic memory is that it will automatically lead to discounting future value based on the number of episodic transitions that are necessary to reach the valued memory state. Figure 9A shows the decision between two stimuli where one has an immediate value and the other is only indirectly associated with a value through a number of episodic associations, ranging from none to nine steps. As can be seen, the probability of choosing the immediate value (or reward) increases with the length of the associative sequence needed to find the value of the alternative choice. The main reason for this is that a longer episodic sequence with a value at the end will update its corresponding accumulator less often and will consequently be less likely to win. However, the shifting of attention also influences the process since it will be more likely to interrupt a longer episodic sequence before it reaches a state with value.

Unlike a planning process, there is not necessarily any systematic evaluation of different possible future action sequences. The decision process aims at making a decision between alternatives that are available here and now. However, the mechanisms proposed here are compatible with such a more systematic search through memory.

The episodic recall mechanism can also be used to select a delayed larger reward over an immediate smaller reward. Figure 9B shows an example with stimulus A having value 0.9 and stimulus B having value 1. When both values are available immediately, the model will mostly select stimulus B, but as the number of memory transitions needed increases, the model will become more likely to select the immediate lower reward. This is entirely a system property of the model as there is no explicitly set discount factor.

### 3.5. Top-Down Feedback to Attention System

**Figure 10:**
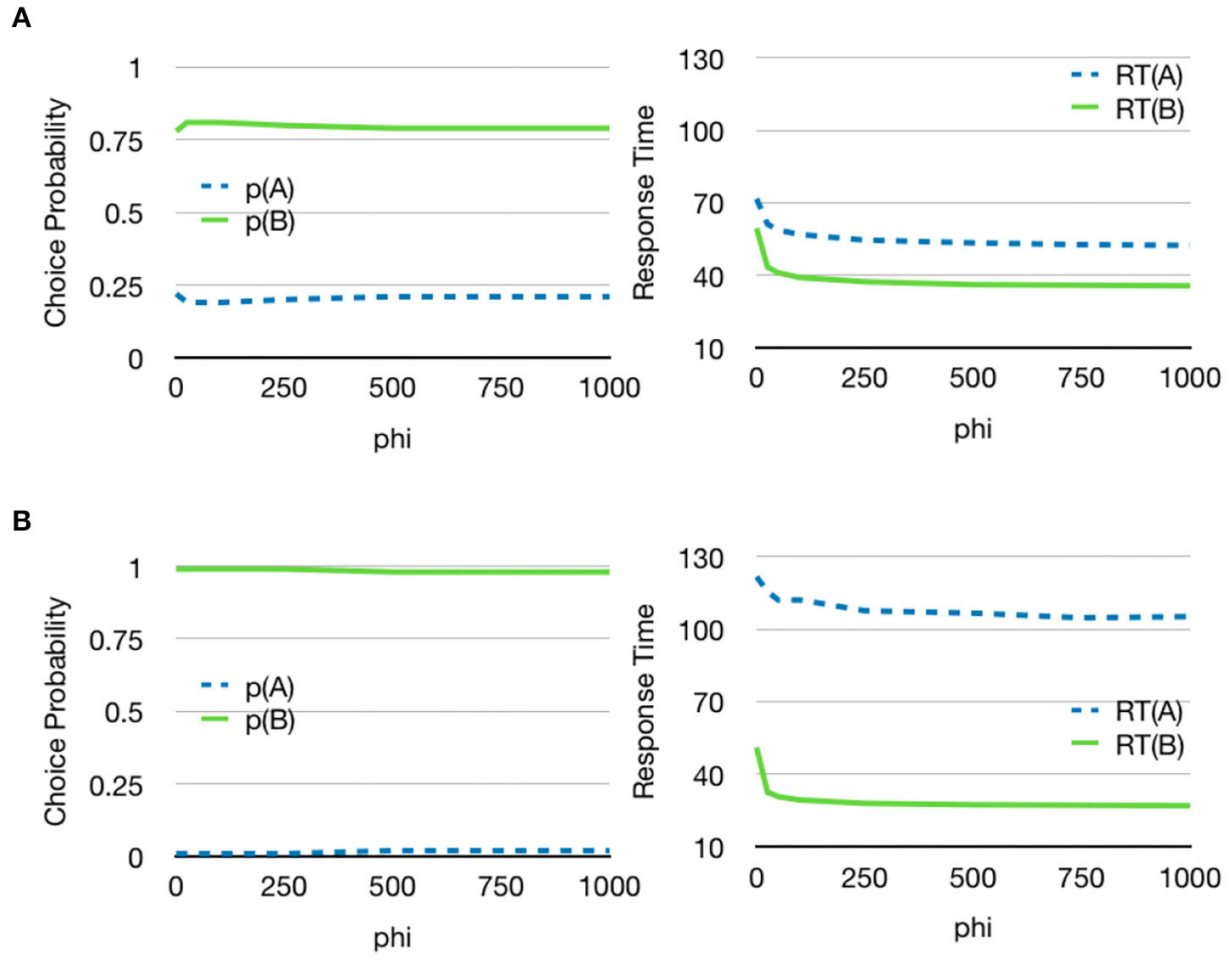
Decreased reaction time with top-down feedback from the decision component. **(A)** The two stimuli had value *V*(*A*) = 0.4 and *V*(*B*) = 0.6. The value of φ was moved from 0 to 1,000 and as can be seen, a higher influence from top-down attention decreased the reaction time as more time is spent looking the alternative that will eventually be chosen. There is a negligible effect on the choice probabilities. **(B)** A similar effect can be seen for a larger difference in values (*V*(*A*) = 0.2 and *V*(*B*) = 0.8). See Supplementary Material for additional parameters.

The model also includes top-down feedback from the decision process to the attention system. When the gain of this feedback is increased, choices are faster and the system will look more at the alternative that will finally be chosen (Figure 10). This is an effect that has also been found in empirical studies (Gidlöf et al., 2017).

## 4. Discussion

We have presented a new system-level model of decision making that combines components for attention, perception, semantic, and episodic memory with an accumulator stage that adds up value over time until a choice can be made. The model has a number of attractive properties:

When perceptual states are directly associated with value through the memory component, the model reduces to the value function of a reinforcement learning system (Sutton and Barto, 2018), or critic of an actor-critic architecture (Joel et al., 2002). Since we do not model different actions here, the system is assumed to interact with whatever object is selected. The accumulator and decision mechanisms thus implement a selection policy over the different perceived objects in the environment.

The model explains how attention, memory and decision making interact through the use of spatial indices that bind the different processes together. A single alternative is processed at a time in the flow from perception to valuation, while the spatial attention component keeps track of the different alternatives and makes sure that their values are separately processed by the accumulators.

Another property of the model is that is explains how different kinds of memory structures can be used to support decision making and how different kinds of associations with different time constants can all contribute to a decision. An unexpected consequence of episodic associations is that its interaction with the accumulator will cause future values to be discounted. The model suggests that the discounting of future value is not governed by a decaying process during learning but is the result of episodic memories that are slower to influence the accumulators the more memory transitions are made before reaching a valued state. Shorter episodic sequences will thus have an advantage over longer sequences if they lead to the state with the same value. There is thus no specific discounting mechanism in the model. Instead, discounting is a consequence of how the memory, value and accumulator components interact. This leads to the prediction that an alternative that contains less details and thus produces less transitions should be favored over an alternative that produces many transitions given that the values are the same. As far as we know, this has not been studied empirically.

We can compare the decision mechanism proposed here to two other main alternatives that have been proposed in the literature. In classical learning theory, stimulus-response chains are learned at the goal and gradually extended to a sequence leading from start to goal. By higher order conditioning the reward at the goal is propagated backwards in the sequence during training, setting up a goal gradient toward the goal (Hull, 1932). The value of each association is assumed to be weaker the earlier in the sequence it occurs. This is the idea that is also used in modern reinforcement learning (Sutton and Barto, 2018). The reinforcement is discounted at each step to make the system prefer shorter sequences from start to goal. In its simplest form, reinforcement learning models only learn when they receive primary or higher order reinforcement and learning is tied to a specific reinforcing goal.

The second alternative is to learn a cognitive map in the form of associations between states (or locations). One such computational model was proposed by Schmajuk and Thieme (1992). Unlike in traditional reinforcement learning, the goal gradient is here set up dynamically by activating the goal state and the activity is then propagated to other states depending on how closely associated they are with the goal. This is similar to the classical grassfire algorithm for path planning. An advantage of this type of model is that the learned model is independent of any particular goal and that it does not need reinforcement to learn. It is thus suitable for explaining latent learning (Tolman and Honzik, 1930). This method is similar to backward search in state space planning (Ghallab et al., 2004).

What we have proposed here is a third alternative that focuses on choosing between alternatives that are available here and now. Instead of using a learned gradient of discounted value as in reinforcement learning, or a gradient set up by a specific goal as cognitive map models, the process starts with scanning the different alternatives and works toward a state with value (such as a goal state) through associating the properties of the observed alternatives with value of other memory states that have value. This method is similar to forward search in state space planning (Ghallab et al., 2004) but is much less systematic since it depends on the exact chains of associations that have been previously learned. In the future, we want to investigate how efficient this method is as a planning and problem solving mechanism. This will require additional components to control metaparameters, such as the level of noise in the both in the memory and accumulator components.

In the simulations, we used a binary model of object salience, but the model is compatible with a more developed saliency map approach where target object are initially selected based on the visual salience. Such bottom up salience can interact with top down stimulus bias from the accumulator component to select which objects to consider.

There are a number of venues for future research. In the future, we want to analyze the model from a learning perspective to see how it compares on reinforcement learning tasks. This entails setting up simulations where the model is allowed to interact with an environment where it can learn semantic, episodic and value associations. It would also be interesting to further analyze the properties of the implicit discounting that occurs as a result of the episodic associations.

Another extension is to include additional mechanisms that were not included in the current version of the model. This includes adaptive gain control for memory retrieval (Mather and Sutherland, 2011) and value accumulation (Aston-Jones and Cohen, 2005). It would also be possible to include a number of additional associative connections. One can add a number of possible interactions between the different components that were not included in the present model but have been explored elsewhere. These are drawn in gray in Figure 1. The memory component could influence perception and produce priming effects. The value component could influence memory recall and indirectly also the perceptual processes (Billing and Balkenius, 2014). Associations between value and spatial attention could bias the search process to particular locations and interactions between memory and spatial attention may enhance memory storage and recall (Balkenius et al., 2018). Mechanisms for context processing could also be included to make the associative process more efficient and goal directed.

A final line of development will be to adapt the parameters of the model to empirical data to see how well it can explain more quantitative aspects of decision making. Even though each of the components of the model are relatively simple, there are still a large number of parameters that interact to produce the different properties of the model. It will be interesting to see if there is a consistent set of parameters that can reproduce the empirical results.

In summary, we have presented a novel system-level model of decision making that describes how components for attention, perception, memory, evaluation, and value accumulation can interact in a natural way. The model describes how semantic and episodic memory can be combined with a decision mechanism to choose between alternatives.

## Data Availability Statement

The source code for the simulations reported in the study is available at: https://github.com/ikaros-project/ikaros.

## Author Contributions

CB, TT, BJ, AW, and PG planned the paper, the theoretical framework, and wrote the paper. CB, TT, and BJ implemented the computer simulations. All authors contributed to the article and approved the submitted version.

## Conflict of Interest

The authors declare that the research was conducted in the absence of any commercial or financial relationships that could be construed as a potential conflict of interest.
