# Supplementary figures and images for: The Missing Link Between Memory and Reinforcement Learning
Source: Front Psychol. 2020 Dec 10;11:560080. doi: 10.3389/fpsyg.2020.560080 (PMC7758424; doi:10.3389/fpsyg.2020.560080)

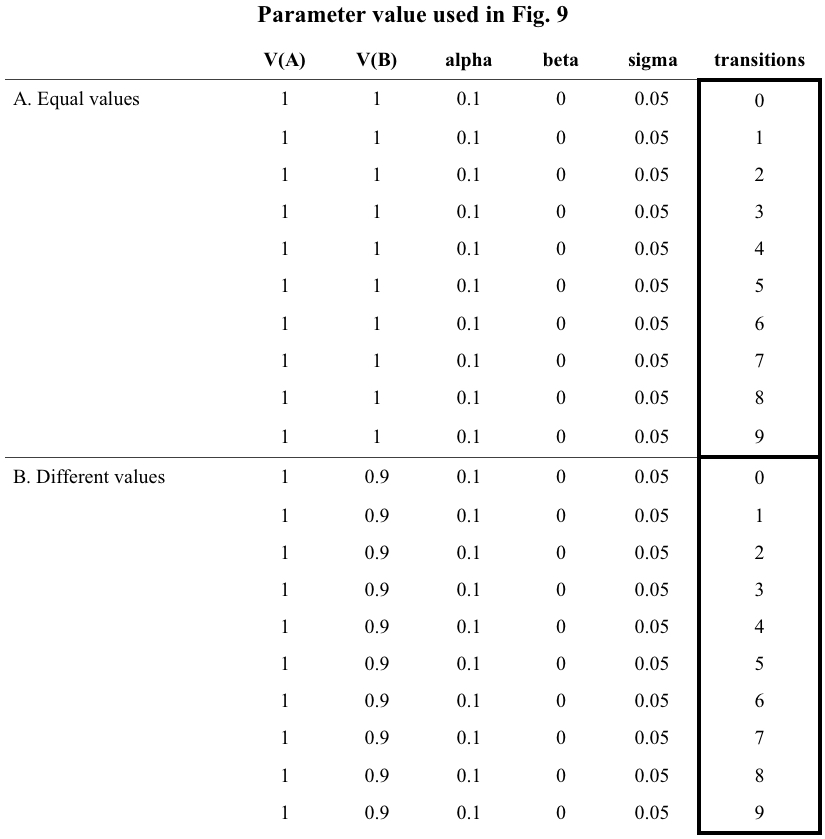

Supplement: Supplementary file 1 [file Image_1.JPEG]

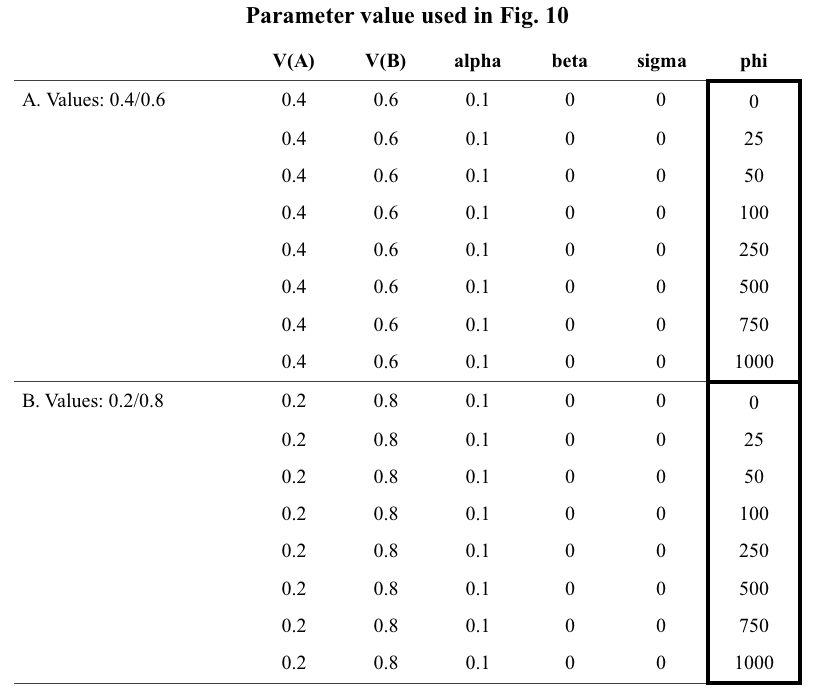

Supplement: Supplementary file 2 [file Image_2.JPEG]

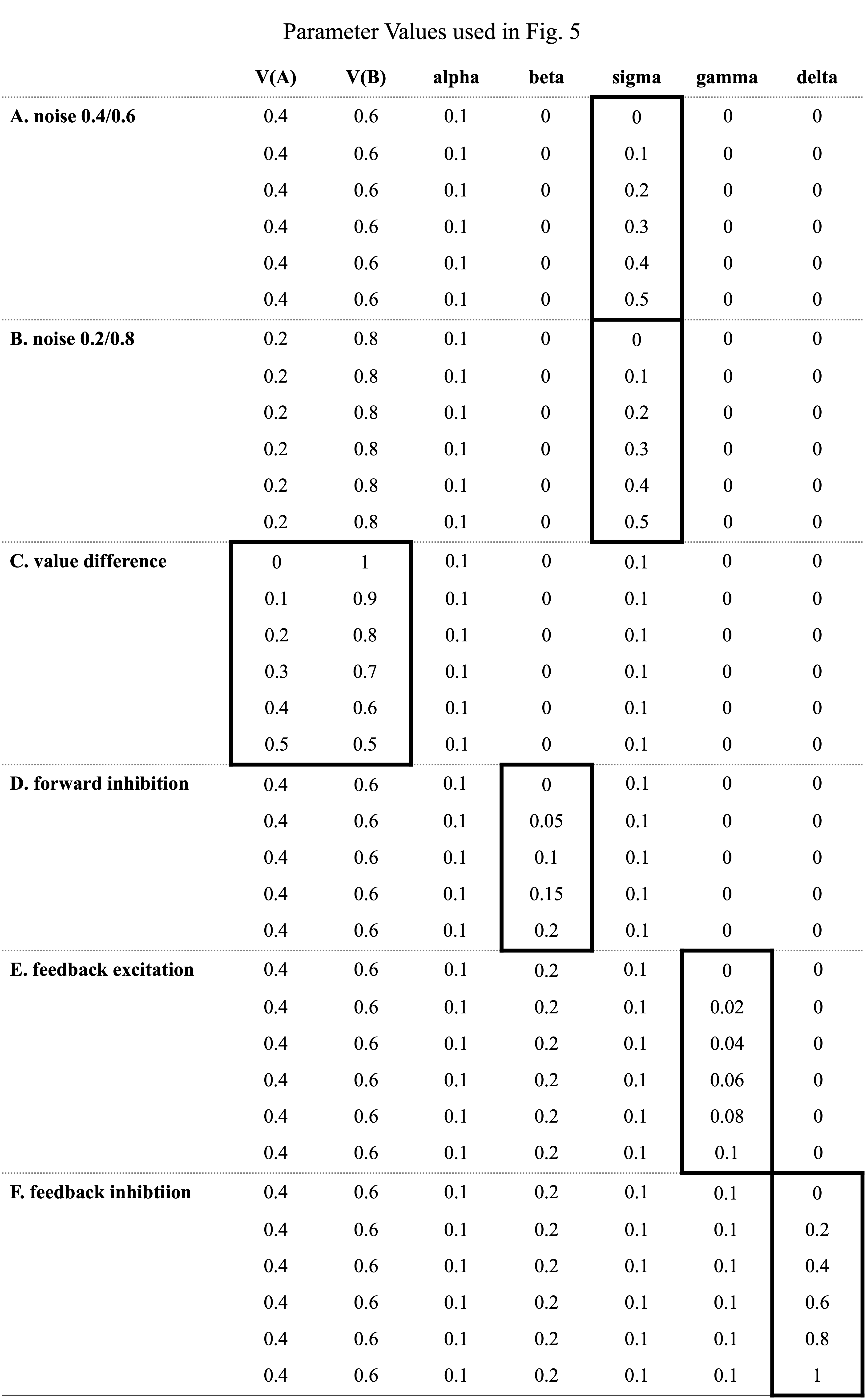

Supplement: Supplementary file 3 [file Image_3.JPEG]
